# Collaborative use of a 3D anatomy platform to motivate and enhance anatomy learning in first‐year online medical sonography students

**DOI:** 10.1002/jmrs.848

**Published:** 2024-12-13

**Authors:** Michelle Fenech, Nadia Mead

**Affiliations:** ^1^ Central Queensland University, College of Clinical Sciences, Health, Medical and Applied Sciences Brisbane Queensland Australia; ^2^ Department of Medical Imaging Royal Brisbane and Women's Hospital Brisbane Queensland Australia; ^3^ Central Queensland University, College of Education and the Arts Cairns City Queensland Australia

**Keywords:** Anatomy education, medical image recognition skills, medical imaging, online learning, social playful learning, ultrasound anatomy

## Abstract

**Introduction:**

Sonography students require a deep understanding of structural anatomy, including where structures sit relative to one another. Additionally, they need to learn the complex task of identifying structures on medical images including X‐rays, computed tomography, magnetic resonance imaging and ultrasound imaging. Anatomy can be taught online, but learning can be challenging for students. Online three‐dimensional (3D) anatomy platforms aid student learning, but research investigating how to use them effectively when teaching is scarce. This project evaluated the impact of incorporating a three‐dimensional (3D) online anatomy platform into teaching relative structural anatomy and assist sonography students when learning how to recognise structures on medical images.

**Methods:**

Sixty‐one first‐year sonography students within an articulated undergraduate and postgraduate medical sonography programme in Australia, studying anatomy online participated in this mixed methods study. The impact of using a 3D online anatomy platform on their conceptual 3D anatomy understanding of relative anatomy, development of medical image recognition skills and their learning experience was assessed via a Qualtrics survey.

**Results:**

Students who used this platform collaboratively enhanced their relative anatomy understanding and developed the skill of identifying structures from medical images. The scaffolded use of this platform generated enquiry discussions between teachers and students and made learning anatomy online a social and enjoyable experience.

**Conclusion:**

Collaborative and interactive scaffolded use of 3D online anatomy platforms can motivate and encourage student questions and discussions, enabling social connections and enhancing their learning experience. Student enquiry skills were developed, and the more complex task of identifying structures from medical images was made achievable.

## Graphical abstract

Studying anatomy online, particularly learning how to identify structures from medical images is challenging. Using a 3D online anatomy platform in a scaffolded manner, enhanced relational anatomy learning and understanding in first‐year medical sonography students, who undertake their foundational learning online. Learning relational anatomy and how to identify structures from medical images in an online environment is challenging. The scaffolded use of an interactive 3D anatomy platform allowed learning to be more active and generated discussions to enhance spatial 3D anatomy understanding, which improved connections between students and made learning playful, social and fun.

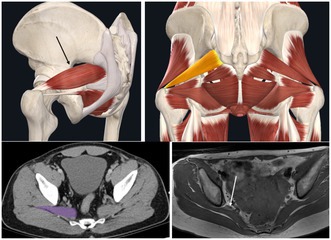

## Introduction

Anatomy is core foundational knowledge for pre‐clinical learning for medical, nursing and allied health professions.^1^ The level of anatomical knowledge required is profession‐dependent and must be tailored to meet the specific needs of the discipline.^2^ Sonography students are required to learn and understand where detailed anatomical structures sit relative to each other and how to identify structures from medical images including X‐rays, computed tomography (CT), magnetic resonance imaging (MRI) and ultrasound imaging. The translation of complex three‐dimensional (3D) anatomy knowledge to allow medical image recognition skills is required early in their learning journey. Designing a curriculum that contextualises learning, develops this complex 3D understanding of anatomical relationships and enables identification of structures from medical images can be challenging. This is made even more difficult when students undertake anatomy learning online.

Learning anatomy online, when appropriately facilitated by teachers, has the advantages of flexibility of pace, place and time, providing improved accessibility and equitability for a diverse and geographically separated student body.^3^ It can be challenging designing and planning learning activities to make online anatomy learning an active and interactive process which encourages the development of building long‐term memory and deep understanding.^4^, ^5^, ^6^ Furthermore, fostering an online learning community, where conversations and interactions with the teacher and between students occur regularly is also a challenge. Ensuring students are connected when learning is important because how a student feels when learning, including having a sense of belonging, can impact their engagement with studies and eventual success.^7^ What is currently lacking in the literature, are studies investigating teaching practices to foster 3D anatomy conceptual understanding to allow the identification of structures from medical images when students are studying solely online. Lacking also from the scholarship are studies that show how to ensure a positive learning experience for those students.

3D anatomy tools can be utilised to assist students learning anatomy online to develop spatial anatomical knowledge and encourage active and collaborative learning.^8^ The use of online anatomy tools must be appropriately facilitated by teachers to ensure relevant scholarly use, active student engagement and the development of correct student understanding.^9^, ^10^, ^11^ In the case of sonography students, this anatomy understanding needs to be transferable to medical image interpretation. This study investigated the incorporated and scaffolded use of a 3D anatomy learning platform, Complete Anatomy (3D4D Medical, CA, USA) into the curriculum design of a first‐year subject (unit) within a dedicated articulated undergraduate and post graduate medical sonography programme where medical sonography students learn anatomy and image recognition skills entirely online (only sonography students enrolled in this unit). The study aimed to determine whether its use:

1. Enhanced relative and 3D structural anatomy understanding to allow the correct recognition of structures from medical images including X‐rays, computed tomography (CT), magnetic resonance imaging (MRI) and ultrasound and
2. Motivated active, collaborative learning of online students to improve their learning experience.

## Materials and Methods

### Study design and participants

A mixed methods study was conducted at a large regional, multi‐campus university. One hundred and ninety eight first‐year medical sonography students enrolled in a dedicated four‐year articulated bachelor and graduate diploma medical sonography course were invited to participate in the study. Students were enrolled at five campuses, widely geographically distributed around Australia (Brisbane, Sydney, Melbourne, Perth and Mackay). The student participants were enrolled in a subject called ‘Relational Anatomy and Image Recognition’ with learning conducted entirely online over a 12‐week learning period. The subject was only offered to sonography students, and they were concurrently learning physiology in other subjects. Sonography students are required to successfully complete this subject as pre‐requisite learning before moving to the second year of the sonography programme where they start learning practical sonographic skills.

### Survey

In 2021 the curriculum of this subject was adapted to incorporate the use of an online 3D learning resource ‘Complete Anatomy’ (3D4D Medical, CA, USA) via the university purchase of a licence for student and teacher use. An anonymised Qualtrics survey with closed and open‐ended questions was designed and students were requested to complete the same survey at five points during their learning period (weeks 3, 5, 8, 12 and 14). The last survey was completed at the cessation of the learning period, after associated assessments were completed and grades and feedback were released. All five surveys were required to be completed by participants. The timing of the surveys was selected to investigate whether students found the resource beneficial for learning a variety of different body regions and throughout their learning period. Data were accessed and analysed at the completion of the study period.

Human Research Ethics Committee approval to conduct the study was obtained from the Central Queensland University (CQU) Human Research Ethics Committee (HREC) (# 23041). Student participants were invited to complete anonymous surveys, with informed consent gained prior to survey commencement. Open‐ended survey questions included:

1. Did using the platform help you develop an understanding of anatomical structures and their three‐dimensional relationships? How?
2. Did using this platform aid you to correctly identify structures from medical images? How?
3. Did you actively use this platform in study groups with your peers? If so, did it help generate questions and discussions to improve your learning and how?
4. Did using this platform motivate your learning of anatomy during this subject? Why?
5. If you attended peer study groups, did using this platform help you meet other students and make connections with others? How?

### How the anatomy tool was incorporated into learning

To guide student learning, pre‐recorded weekly lectures were provided to unpack anatomy content for students which placed learning in context. Synchronous interactive, teacher‐facilitated online tutorials were provided twice a week where systematic thinking approaches were modelled.^12^ Students were guided to use the 3D online anatomy model to identify structures and describe detailed structural relationships. Online anatomy models were rotated in real‐time simultaneously by both the teacher and students; structures were able to be viewed from different directions and angles, and layers of different tissue types to be subtracted and added to a base image so the depth relationships of structures could be demonstrated, discussed and appreciated. Series of medical images (from multiple modalities) were viewed collaboratively, the imaging planes discussed and structures identified. A systematic approach to identifying structures was modelled, starting with more obvious and larger structures such as bones, muscles and organs which act as landmarks, to signpost the positions of smaller and more detailed structures such as neurovascular structures.^12^

In addition to teacher‐led tutorials, peer study sessions were facilitated, where higher year‐level students from the same programme were engaged to mentor and guide first‐year students in online sessions. Together students used the 3D platform, providing interactive playful learning sessions, ensuring learning was tailored and contextualised for the sonographic discipline. Students were also encouraged to establish and run their own online first‐year student study groups to collectively use the 3D resource together, to further aid their learning.

### Data analysis

From the survey results, descriptive statistics related to closed‐ended questions were calculated. For open‐ended questions with qualitative answers, thematic analysis using iterative enquiry was conducted separately by two researchers to identify the perspectives and experiences of the student participants.^13^, ^14^, ^15^ Initially useful quotations were selected and keywords identified. Coding was conducted to organise data into meaningful categories via the use of manual codebooks.^16^ Themes were identified and constructed separately by both researchers and then compared, reviewed and refined.^17^

## Results

Out of the 198 students enrolled in this subject, 61 participants each responded to all five surveys. Since 2020 and the COVID‐19 pandemic universities have seen a decline in online response rates regarding student evaluation of learning and teaching and surveys.^18^ A 31% response rate to this survey was considered reasonable.

At the start of the learning period, 53% of students were excited to learn anatomy and 26% were concerned about learning anatomy online. Throughout the learning period, 98% of respondents used the 3D online anatomy platform by themselves and with others. 64% of respondents formed their own first‐year peer study groups, used the resource together, asked questions of one another, problem‐solving collectively where structures sat on the 3D anatomy models and on medical images.

Qualitative analysis identified three main themes which included: 1. Peer‐peer learning was encouraged 2. Interacting with the resource deepened conceptual anatomy understanding 3. Collaborative problem‐solving was social, enjoyable and motivated learning. The themes are outlined and unpacked below.

### Peer‐peer learning was encouraged

The 3D tool provided a common ground for initiating conversations for students and discussions with others. Peer discussions allowed students to use the resource together and clarify their understanding. Typically students reported in response to open‐ended questions:

You could use it to hold little study sessions with others anywhere… (they) kept me motivated… were fun and I found I would better understand where things sat relative to one another when I heard other students explanations or I explained the relationships to others. (S39)

### Interacting with the resource deepened conceptual anatomy understanding

Students reported that rotating the anatomy models online as a group online and collectively identifying structures from a series of CT, MRI (coronal, axial and sagittal) and ultrasound images, allowed the relevance of relational anatomy knowledge to be gained. Interacting with the resource required students' active participation in learning. Additionally, questions regarding knowledge checks could be asked and answered which aided the transference of theoretical knowledge to allow image identification skills to be developed outlined by representative student quotes:

It was great to use when you don't fully understand where a structure sits in relation to another. We would collectively work out the relationships of structures and work out how we had to describe the relationship using appropriate terminology, when we had to explain it to someone else out loud. (S14)

It was really helpful to see where the structures sit visually and then transfer that into medical images…we could do this together which was satisfying. (S27)

Students enjoyed interacting with the online 3D platform, and with each other, virtually adding and subtracting layers of tissues and structures to develop their understanding of the relationships of structures, together creating their own understanding citing:

I loved being able to add/subtract layers of structures so I could see what sat superficial and deep to other structures. I also found the slice images helpful so I could gain an understanding about an axial view. (S25)

### Collaborative problem‐solving was social, enjoyable and motivated learning

An unexpected theme which emerged from the collaborative use of the 3D online tool was that learning became a social, fun and enjoyable experience, which motivated further learning. Collaborative discussions using the 3D anatomy tool provided the opportunity for progress check‐ins with students regarding not only their learning, but also their general well‐being and how they were coping generally whilst studying. Students typically commented that the check‐ins prevented their feelings of helplessness, frustration and isolation which they had experienced with online learning in other first‐year teaching terms:

I felt a sense of belonging to the sonography course through my anatomy study groups when we used Complete Anatomy, I could talk to other students on a regular basis, and I felt less lonely, and so I looked forward to my study groups with my new friends. (S14)

As the 3D anatomy resource made the online learning experience collaborative, working with other students made it enjoyable for 95% of respondents.

I could easily trace vasculature, which is helpful in understanding where vessels sat on CT images when following CT slices from superior to inferior or proximal to distal. I enjoyed doing this together with other students online. (S17)

When I was looking at a medical image and I became confused, I then went to “Complete Anatomy” and was able to “see” the medical image more in 3D by taking away structures. I was able to understand the ‘depth’ of structures, not apprarent on a 2D medical image… gaining the ah‐ha moment with others was satisfying and I really enjoyed studying anatomy, which I was not expecting. (S3)

As a result of using the 3D anatomy platform, 98% of students recommended its continued use for future medical imaging and sonography students, and across the wider university. All participants who took part in the study wanted continued access to the platform in their higher year levels to further assist their learning.

## Discussion

Anatomy is a visual science. Traditionally, laboratory‐based human cadaveric dissections have been used as teaching tools to support gross anatomical learning; they allow spatial relationships of structures to be appreciated and provide sensory, visual and tactile experiences.^19^ Dissections in labs offer face‐to‐face learning and are often conducted in groups, providing the benefit of generating student peer‐peer discussions which assists learning.^20^ However, anatomy understanding gained via cadaveric dissections can depend on factors related to the learner including visuospatial ability, and ethical, emotional, anxiety and sensory concerns associated with dealing with cadavers.^21^ Many clinicians rated their anatomic knowledge gained from cadaveric dissections as insufficient and not transferable to their clinical health professional role.^4^, ^22^ With less access to cadavers, their associated high costs and ethical considerations, anatomy education is evolving. Anatomy learning is increasingly being undertaken online.^5^ Online learning has the challenges of lack of tactile learning and undertaking manual tasks which can promote active learning. Strategies to overcome these challenges are required.

Learning structural anatomy is a building process, which requires active construction of knowledge.^23^ When teaching foundational structural anatomy completely online, constructive, collaborative, contextualised active and self‐directed learning can be facilitated by the teacher. However, fostering peer‐to‐peer interactions between online students can be challenging.^24^ Digital and online learning can have advantages over conventional classroom learning, as they allow for technologically enhanced, innovative, flexible, accessible and equitable learning for learners geographically distributed. Conceptual thinking regarding anatomy needs to be developed during the learning process, and this can be difficult for learners not to discuss concepts with others. Teachers need to consider how they convey subject knowledge, but also challenge students to collaboratively produce and create understandings.^25^

In this study, regular teacher‐facilitated online tutorials where the teacher and the students simultaneously used the 3D online anatomy platform, allowed for consistent communication between students and their teacher. The curriculum design was based on Vygotsky's zone of proximal development.^26^ Students were guided and stepped through the process of first ‘the learner cannot do’; using the 3D anatomy platform online helped students develop a spatial understanding of where structures sit in the body.

Next, learners were shepherded to ‘the learner can do with guidance’; they were shown how to identify a structure, relative to smaller structures, both on the online 3D platform and then on medical images. Problem‐solving strategies to identify larger and then smaller structures on medical images which were gained from contemporary clinical practice, in three planes, were modelled by the teacher and then by the student. The reason for imaging was discussed to contextualise learning. Both normal and abnormal medical images were presented to allow students to appreciate why and how medical imaging can aid a patient diagnosis and inform medical treatment algorithms.

Eventually, students were ushered to the step of ‘the learner can do unaided’ where they worked with each other to identify and follow structures (such as vessels) through a series of medical images (for example abdominal CT images from superior to inferior) allowing students to effectively use their working memory.^27^ Peer‐peer learning allowed them to discuss, clarify and correct their thoughts. Students eventually achieved the goal of independent practice from experiential learning.^28^, ^29^

It is important that student fascination with anatomy is evoked, to encourage lifelong learning.^30^ This is particularly important for medical imaging health professionals, where ongoing professional development is essential for continued clinical practice. Learning is a process, rather than an outcome, grounded in experience, linked to the personality of the user.^28^ The guided practice approach to learning anatomical relationships in 3D, discussing with others and reflecting on this, and then transferring knowledge to allow identification of structures from medical images enabled students to develop conceptual thinking skills.^31^, ^32^, ^33^

The consistent weekly use of the 3D online anatomical platform via tutor and peer‐facilitated environments reinforced relative anatomy concepts as per the threshold concept of learning.^34^, ^35^ New and previously inaccessible ways of thinking were gained.^34^, ^35^, ^36^, ^37^ This facilitated transformative student learning and for knowledge to be continuously created and re‐created through transactions between the learner, their environment and their experiences.^38^, ^39^, ^40^, ^41^, ^42^, ^43^ Additionally, as learning was contextualised, students identified that the knowledge they gained was going to be well integrated into their future learning and eventual professional practice as a medical imaging professional.^34^ The culture of positive authentic learning developed improved student engagement and enthusiasm for their programme of study.

Learning environments that motivate learning and facilitate interactions with teachers and peers are essential, but can be challenging to foster online.^44^ Completing first‐year studies online, can be an extremely lonely experience for students, particularly school leavers, who are used to interacting face‐to‐face with teachers and peers on a regular basis.^45^ Students can be easily distracted from study and anxious about approaching their teacher or peers in an online environment.^46^ Scheduled regular interactions and discussions using the online 3D anatomy tool provided engaging, interactive, regular catch‐ups.

The online 3D anatomy tool became a catalyst to empower students to enhance their autonomy and promote learning and growth.^25^ It provided a common topic for discussion within peer‐to‐peer interactions, which not only generated critical questions, and developed problem‐solving skills, by having to identify structures on medical images, but also enhanced their social experience.^46^, ^47^ The 3D anatomy platform, therefore, allowed learning anatomy to be social, playful and an enjoyable experience, rather than a laboursome task.^48^ By having access to a resource to use together online, students gained a sense of connectedness to each other and a sense of belonging in their programme of study.^41^ Hence, integrating technologies and online tools into pedagogy, when used interactively and effectively can improve dialogue between teachers and students and improve the student learning experience.

Limitations of this study included that it relied on students to report on their development of correct understanding. More detailed qualitative information related to the numbers of students in peer study groups, and how they were facilitated was not obtained. Future studies could focus explicitly on well‐being and learning motivation and make this transparent in the survey questions.

## Conclusion

The scaffolded use of a 3D anatomy platform, through teacher and peer‐facilitated online learning activities, generated questions and discussions between peers enhancing student spatial understanding of 3D relative anatomical structures. It also enabled transferable learning, allowing correct identification of structures on medical images, a challenging concept to grasp. Importantly, the interactive use of this online resource allowed learning to be active and playful, which motivated learning by making it social, interactive and fun thereby enhancing the learning experience of students.

## Funding

This work was not funded by any grant‐awarding bodies.

## Conflict of Interest

The authors declare no conflict of interest.

## Data Availability

The data that supports the findings of this study are available from the corresponding author upon reasonable request.
